# Gleanings from Our Exchanges

**Published:** 1873-03-01

**Authors:** 


Fibroid Tumors of the Uterus.—Alfred Meadows, M.D., London, England (*Am. Jour. Obstetrics*) in his "Remarks on the Diagnosis and Surgical Treatment of Fibroid Tumors of the Uterus," says that, having determined the situation of the tumor and its interstitial character, one is justified in attempting the removal of these tumors, even though they be not intra-uterine or submucous, but are situated in the substance of the uterus itself, provided a proper canal be inaugurated. His plan is, first of all, to prepare the passages for the expulsion of the growth, and, secondly, to detach the tumor from as much of its surroundings as possible, so that, by making of it a foreign body, nature may aid in its removal, as she would in the case of a dead fœtus or a mole-pregnancy, or even a uterine polypus. Lastly, when nature has been given fair play, the ecraseur should come to the rescue, and remove at once what might otherwise be the work of many months or years. He had recently under his care a case in which the tumor was completely imbedded in the substance of the uterus, so much so that the os was not dilated in the very least; and he had the satisfaction, after three or four operations, of completely removing the tumor, which was of the size of a small cocoa-nut. The patient is now perfectly well.

At the date of writing he had two other cases of the same kind, but in both the tumor was much larger. He had commenced with the same plan of treatment in these cases, and he had every reason to believe that a cure would be effected.

The first step in the process is to prepare the passages for the removal of the tumor. For this purpose he recommends free division of the cervix uteri in one or more directions. The next step is breaking with the finger through the capsule, and little by little detaching the tumor from its bed. During the intervals efforts should be made, by the administration of ergot, borax, cinnamon, and other so-called oxytoxics, to secure contraction of the uterus, so as to favor nature's method of expulsion. Galvanism is also another agent of great power in this respect; and a firm bandage is of service in cases where the tumor is large and projects well into the abdomial cavity.—*Med. Record.*

Extensive Drainage in Anasarca.—The Boston *Med. & Surg. Journal* describes a novel treatment of anasarca employed by a surgeon in Prussia. Twenty-five hypodermic canulæ were introduced through the skin, in different parts, and left there three days, during which time twenty quarts of fluid were discharged. The fluid was conducted into vessels through elastic tubes. No inflammation or other unpleasant results followed.

### Gleanings from Our Exchanges.

### ON THE ACTION OF CABBAGE LEAVES ON ULCERS AND DISEASES OF THE SKIN.

The following is from *The Doctor*, February 1st., 1873 :

Dr. Blanc has published, in some late numbers of the *Revue de Therapeutique*, a very long memoir on the above. Space would fail us to reproduce it, but we believe it would interest our readers if we introduced to them at least his conclusions.

Moreover, he works with an external therapeutic agent so easy to obtain, that its name alone is sufficient to arrest the attention of medical men.

This memoir, Dr. Blanc says in closing, establishes sufficiently how useful the cabbage leaf is in many cases of cutaneous disease, by actions new, in a practical as well as a theoretical point of view. These facts have their laws. I will formulate them, as I understand it, in the following propositions :—

1. The cabbage leaf excites and augments suppuration, or the secretion of ulcers, ulcerations, vesicles, and pustules. It has the same action on the integuments affected by an erysipelatous or furunculous inflammation, but recovers tissues in a morbid condition.

2. This augmentation of suppuration is constantly followed by an amelioration, and often by a cure. It is the condition necessary to the result ; and the property of the leaf, which determines this result, is an indirectly curative property.

3. This property does not consist in any principle which the leaf yields for absorption, but rather in an affinity which the leaf has for the vitiated secretions.

4. The leaf exercises this affinity on open ulcers, or on ulcers covered by a thick or thin scab or crust ; it exercises it on the thickened epidermis, or where it is converted into thickened rind-like membranes ; in simple or confluent variola, throughout mortified tissues, through the integuments, whether inflamed or non-inflamed, but recovers tumors capable of absorption.

5. When the tegumentary affection is wide-spread or general, the action of the leaves, on the parts where they are applied, benefits the whole disease.

6. The matter in the parts not covered by leaves is absorbed, and at once directed under the leaves to be immediately excreted at that part.

7. Treatment by the leaves of a suppurative affection prevents re-absorption and consequent pyæmia.

8. The cure obtained by this means is more complete and certain than by any other, because it is brought about only when the cause and products of disease are eliminated from the system.

9. This mode of treatment is in perfect harmony of action with the *vis medicatrix naturæ*. This essays in skin diseases to eject from the system their cause and effects, whilst the leaves aid this action.

10. The cure of an ulcer by the leaves, however wide-spread and long-standing it may be, is without danger, and relapse is very rare.

11. The cicatrices obtained by the leaves are remarkable for their small degree of deformity.

12. Small-pox, measles, and scarlatina, treated by applications of the leaves, have few or no sequelæ ; *e. g.*, phthisis is not to be feared.

13. The cabbage, which is employed externally, and in the natural condition, cannot, at the time, yield to the organism any principle capable of neutralizing the cause of a malady and destroying its effects ; and since the cure operates by suppuration and secretion, induced and stimulated by the leaves, we must conclude—

1st. That in case of a cure the cause of the disease has been excreted by secretion or suppuration.

2d. That some diseases have for their immediate cause a vitiation *sui generis* of the fluids.

3d. That the *vis medicatrix* proceeds to the cure of these diseases by driving the vitiated matters towards the tegumentary covering, where it spreads them, eliminating them afterwards by vesicles, pustules, or the excretory vessels on the inflamed or ulcerated surfaces.

14. These operations take place in a diseased body; they are then diseased functions—functions which have the integuments for organs ; the integuments modified by inflammation, either simple, vesicular, pustular, or serous.

15. The leaves are the auxiliaries of this function; they attract it out by their affinity for vitiated fluids.

16. This elimination accomplished, the means which have served for it, I should say the modifications of the integuments, being no longer required, disappear ; they are cured.

17. This mode of cure, this treatment, I would call a cutaneous depuration.

J. M. Scott, M.D.

### TREATMENT OF SYPHILIS IN THE HOSPITALS OF VIENNA.

Dr. Hermann gives, in the *All. Wien. Med. Zeit.*, this account of the treatment of syphilis in the Wieden Hospital of Vienna, and other general hospitals of that city. In the Wieden hospital, of which he is physician, no mercury is used, and he claims, firstly, that at least a third of the time is saved by this method of treatment as compared with that by mercury; secondly, that the cost of patients is one-third less; and, thirdly, that the mortality from syphilis is much smaller under the non-mercurial than under the mercurial treatment; fourthly, he contends that the non-mercurial treatment has a notable influence on the lowering of the number of syphilitic patients. Firstly, the most famous representatives of the treatment of syphilis without mercury, Fergusson, Guthrie, Rose, Hennen, Thompson, Listor, Fricke, Bennett, Syme, Drysdale, and others, have found the duration of the treatment of syphilis without mercury about a third shorter than with mercury; and Desruelles thinks that syphilis does not last half as long when no mercury is used. "Of special interest are these percentages which I collected from the public registers of the year 1857, of the 'Allgemeine Krankenhaus,' and the Wieden hospital, where I carry forth the anti-mercurial treatment as a principle. Among those patients (venereal) whose stay in hospital before cure was two weeks, there were 8.66 in the former hospital, and 26 per cent in the Wieden hospital; of those who staid one month there were 25.11 per cent. in the former, and 49.86 in the Wieden hospital; of those who staid two months there were 24.18 in the former, and 13.40 in the Wieden hospital; whilst those who staid three months before cure in the Allgemeine Krankenhaus, were 16.25 per cent., and only 7.50 per cent. in the Wieden hospital.

"Likewise, we may conclude from the above comparison, as to mercurial treatment in the Wieden and other hospitals, that the average duration of mercurial treatment is about sixty days, whilst that of non-mercurial is from thirty to forty days. The senior physician to Inquistenspital, of Vienna, Dr. Edler von Ferroni, arrived at similar results. In the year 1870, there were 244 males, and 99 females treated for venereal disease. Of these, 105 were cases of gonorrhœa, 185 ulcers, and 53 were cases of skin and other affections. The treatment was anti-mercurial, and the average duration of treatment, before cure, was 26.5 days. Of the special forms, the length of treatment in gonorrhœa was 20.4 days; in simple ulcers, 16.9 days; and in cases of gonorrhœa and ulcers with secondary complication, 52.7days. The chief physician of the K. K. Staatsbahn, writes to Dr. Hermann as follows from his yearly report: From 1860 to 1871 there were treated 743 syphilitic patients, of whom 206 were transferred to hospital, and 537 remained who were treated at home (out-patients). The working capability was in most cases lowered by extended, neglected syphilitic ulcers, complicated by buboes, phymosis, paraphymosis, orchitis, or large condylomata. Thus, 537 patients were treated for 9,292 days, during which time they remained unfit for work, so that on an average, 17.30 idle days fell to the lot of each. The whole 537 were completely cured of their venereal diseases."

Dr. Johann Ertl, chief physician in the State Hospital of Gratz, the untiring and courageous champion of the humane ideas of anti-mercurial treatment, has carried on the non-mercurial treatment of the disease for many years, and has obtained the favorable result of an average cure in 32 days.

Dr. Hermann also states that the cost of the non-mercurial treatment is much less than that of the mercurial treatment of syphilis. During ten years, 1861 to 1870, there were treated in the Alservorstadt hospital of Vienna, 20,054, and in the Rudolfstiftung, 6,052 patients, according to the principles of the mercurial school. These patients required 60 days on an average of treatment, and thus 1,566,306 days in hospital, gave an expense of 1,253,088 florins, or 48 guldens a head. In the Wieden hospital, during the same ten years, 14,535 patients were treated without mercury. Their time of stay was on an average 40 days, and the expense, 31 guldens a head.

Thirdly, says Dr. Hermann, it is of higher interest to note the number of persons who have died of syphilis in the three large hospitals of Vienna. According to tables given by the author, it results that, whilst in the General Hospital, one death occurred of 89 syphilitic patients treated, and in the Rudolfstiftung one death took place in 290 treated, in the Wieden hospital there was but one death of 969 syphilitic patients treated from 1861 to 1870. Mercury is the sole cause of the increased mortality in the above hospitals.

Fourthly, Dr. Hermann points out that there is but little increase in the number of cases of venereal disease treated in the Vienna hospitals year by year. From 1861 to 1870, the numbers range between 3,000 to

4,000 annually, for the large hospitals of that city. Two facts are clear, he says, firstly, the number of syphilitic patients holds no pace with the enormous increase of population of late years; and, secondly, the number of venereal cases among women in 1870, (1,599) was the lowest for ten years; which shows that syphilis is falling off in frequency, especially among the prostitute class. The reasons for this, he holds to be, firstly, that a number of patients were called syphilitic who were merely suffering from mercurial poisoning; and one-third of all the prostitutes are now treated without mercury at the Wieden hospital; secondly, until the year 1865, nearly all, and afterwards, the greatest part of the prostitutes under the police, were sent to the Wieden hospital. The proper experience of the patients of the treatment there conducted without mercury, and the observation of other patients, coming from other hospitals with dreadful results of mercurial treatment, caused among the prostitutes a horror and terror for mercury, and made them take all sorts of precautions against contracting the disease, in their power. All of these considerations have taken firm roots in the world of prostitution, and have caused a diminution in the number of cases of syphilis among the class. "Now, the claims of undoubted advantage, the diminution of syphilis in quantity and intensity, which is pointed out in the statistical tables of the State, are undoubtedly the effect of the treatment of venereal patients entirely without mercury in the Wieden hospital. This view is based on 20,000 cases of complete cure during my fourteen years of office in the Wieden hospital, by my attainment of a very small percentage of mortality, and by the confidence of the patients who crowd to the wards of the Wieden hospital the whole year through."

Dr. Ferroni, chief physician to the Inquisitenspital, says: "The number of persons affected with syphilitic disease, which is described as continually increasing, in the General hospital, shows in the Wieden hospital in 1870 and 1871, a pleasantly marked diminution. In 1869 there came 470, in 1870, (when the anti-mercurial treatment was carried out) 318, and in 1871, only 283 patients, with syphilis to that hospital. The cause of this diminution of syphilitic disease, can only be explained by the fact that fewer relapses occur under non-mercurial treatment, and that the larger number of diseased persons in former years, came from the frequent appearance of forms of what were considered to be constitutional syphilis, after the inunction treatment, which were, however, caused by mercurial poisoning."—*The Doctor.*

Sterility from Narrow Os Uteri.—Dr. Pajot (*Gaz. Obst. de Paris*, Jan. 5) mentions the case of a young lady, aged 25, who was in good health and had regular menstruation. She had been married nearly three years to a healthy man, aged 30, and yet had not been pregnant. When the speculum was applied, it was in vain that the os uteri was looked for. At the extremity of the vagina there was noticed a whitish line about two millimetres in length, without trace of any opening, and by means of a stylet it was ascertained that the edges were not agglutinated. The edges of the os uteri were either congenitally or in infancy soldered together, so as to present the appearance of a cicatrix. With all this the young woman affirmed that she had menstruated regularly since the age of fifteen, and that she had never suffered in any way. On this Dr. Pajot examined again with a magnifying glass, and then detected a capillary opening into the uterus. With a very fine stylet he penetrated through the orifice into the cavity of the uterus. Every four or five days a stylet was passed more and more voluminous, and then a dilator used for the lachrymal ducts, so that at the end of three weeks the ordinary dilator could be introduced, and the orifices, after less than six weeks, could be widened up to one centimetre, without suffering to the patient. All attempts at dilation ceased three to four days before menstruation, and for three days after. When the second menstruation approached, sexual congress was permitted, and the menstrual flow did not re-appear. All the signs of pregnancy showed themselves, and soon it was certain. In nine months the lady was confined normally. Dr. Pajot adds, that if gradual dilation be compared with section, all wise surgeons will prefer the former, when it is possible.—*The Owl.*

Ovriotomy an Established Operation.—It is surprising what a change has taken place in the estimate attached to the operation of ovariotomy (or ovariectomy, as it is proposed to call it) since its introduction thirty years ago. For a long time many of the most prominent surgeons shrunk from it and pronounced it murder. We are not aware of a solitary opponent now existing. Statistics give two successful results out of three cases, or sixty-seven per cent.—taking the aggregate of America and Europe. Many capital operations which have been recognized in surgery for centuries, exhibit a greater mortality than this. Ligatures of the great arteries are even more hazardous. Ovariotomy may be regarded not only as an established operation, but the greatest triumph of modern surgery.—*Pacific Med. Jour.*

The Resuscitation of Animation in Newly-Born Children.—Dr. John Gregory, of Manchester, England, calls attention to two opposite conditions which he has found to exist in cases of suspended animation in the newly-born. In the first class the head appears to suffer from a redundancy of blood; and is most common when the head is born some time before the remainder of the body, and the pressure upon the portion remaining in the uterus and the vagina causes an accumulation of blood to take place in the head. This variety is generally relieved by allowing a small quantity of blood to flow from the navel. The second variety is less commonly noticed, and is that in which the reverse takes place. In a breech presentation the head, being born last, is subjected to pressure which empties its vessels and produces anæmia of the nerve-centers of the brain and medulla. Such cases are quickly relieved by placing the child's head downwards, by which posture the return of the blood to the cranium is encouraged. It is his practice in the latter class of cases to allow the infant to hang head downward for about a minute at a time, and employ also friction of the back and nucha. In both varieties the postponement of respiratory movements is attributed by him to disturbance of the circulation in the medulla.—*The Doctor.*

Nitric Acid in Hemorrhoids. — Many cases of hemorrhoidal tumors, especially those which are painful, or which bleed, are best treated by touching them a few times with strong nitric acid. It is an old treatment revived, but one which *we know to be very efficacious.—Pacific Med. Jour.*

Conservative Purpose of Uterine Cancer.—Under this head, Professor King, of the National Medical College at Washington, occupies thirty pages of the *American Journal of Obstetrics*, in an elaborate and highly ingenious essay, more learned than useful we should say. As the article appears in a journal of high authority, we infer that it must possess value, if one could only find it. Our readers will be gratified no doubt by the perusal of the concluding paragaph:

"In conclusion, speaking figuratively, cancer is like the twig that naturally intended to grow in a perpendicular direction, until reaching the towering height of a vertical oak, it becomes, by being bent first to the right, then to the left, then laterally again and again, a crooked, knotted and irregular dwarf. The evolutionary nature of the cancerous organ has, so to speak, been lashed into a pathological fury—goaded to madness—tortured into suicide; like the teased viper, it turns to bite itself with the identical fang naturally designed for its own preservation. Just as a shell, fired at an enemy in a direct line, by unluckily impinging upon a succession of oblique surfaces, may eventually describe a circle and return to burst in the face of the gunner himself; it was fired with the same conservative design of self-preservation, nevertheless."—*Pacific Med. Jour.*

After Treatment of Fibroid Tumors of the Uterus.—After the removal of these tumors, Dr. Alfred Meadows, of London, England (*Am. Jour. Obstetrics*, Aug., 1872.) advocates the subjoined after-treatment: The first thing to do is to secure firm contraction of the uterus after it is emptied of its contents. This is necessary not only to prevent hæmorrhage, but also to avert the occurrence of septicæmia. The latter object will be still further secured by frequent injections of warm solutions of permanganate of potash, carried well up into the uterine cavity. In reference to medicines, he knows of none which are either useful or desirable, except it be opium, and this he regards as of greater value than any or all other medicines put together. He is also very partial to the employment of hot linseed and laudanized poultices to the abdomen in all cases of serious operations upon the uterus where there is a liability to pelvic or peritoneal inflammation.

Treatment of Phagedenic Chancre by Irrigation.—In the treatment of phagedenic chancres, Mr. Hutchinson, of the London Hospital, has for some time, with very good success, used the continuous bath. The patient is ordered to sit in a hip-bath with tepid water for the greater part of the twenty-four hours, a few hours being allowed for sleep. The water is changed as often as it becomes uncomfortably cool. This treatment is kept up until all phagedenic action ceases, and the sore shows a perfectly healthy .surface. It may or may not be necessary to apply nitric acid to the sore in addition to the bath, but the cleansing effect is, in some cases, all that is required.

Mr. Hutchinson attaches great importance to a frequent change of the water in contact with the sore, and lately he has directed the patient to keep a continuous stream of water from an irrigating apparatus playing on the ulcerated surface, while in the hip-bath. By this means every particle of contagious phagedemic discharge is removed as soon as secreted, and the formation of a healthy surface is much accelerated.—*Boston Med. Jour.*

Muscular Contraction a Cause of Fracture.—Geo. W. Norris, M.D., Clinical Surgical Recorder in the University Hospital, Baltimore, Md. (*Rich. & Louis. Med. Jour.*) relates a case of simple fracture of the middle third of the humerus in a mechanic, aged 32 years, caused by violent muscular contraction while throwing a brickbat.

Dr. E. S. Gaillard remarks that Prof. J. M. Holloway recently presented to the class in the Louisville Medical College a case of fracture of the clavicle, caused by throwing an ear of corn.—*Med. Record.*

Equable Climates.—As places of resort for consumptives, California and Minnesota present the strongest claims on the score of equable climates. At San Diego, according to the *Daily Union* of that place, the thermometer has ranged from 60 to 70 at noonday, through the month of January. Even as far north as San Francisco it has not been below 55 at noon, while the average has been 60; nor has it fallen lower than 45 at night. In Minnesota, also, the temperature has been remarkably uniform, that is to say, about 20 below zero. A citizen would have no occasion to put on his overcoat in the former region, nor to take it off in the latter.—*Pacific Med. Jour.*

